# Effects of benzydamine hydrochloride on postoperative sore throat after extubation in children: a randomized controlled trial

**DOI:** 10.1186/s12871-020-00995-y

**Published:** 2020-04-04

**Authors:** Hyung-Been Yhim, Soo-Hyuk Yoon, Young-Eun Jang, Ji-Hyun Lee, Eun-Hee Kim, Jin-Tae Kim, Hee-Soo Kim

**Affiliations:** 1 Department of Anesthesiology and Pain Medicine, Seoul National University Hospital, #101 Daehakno, Jongnogu, Seoul, 03080 Korea; 2 Department of Anesthesiology and Pain Medicine, College of Medicine, Seoul National University, #101 Daehak-ro, Jongno-gu, 03080 Seoul, Republic of Korea

**Keywords:** Benzydamine hydrochloride, Children, Postoperative sore throat

## Abstract

**Background:**

Postoperative sore throat (POST) is a common, undesirable result of endotracheal intubation during general anaesthesia. This study aimed to evaluate the effectiveness of benzydamine hydrochloride (BH) spray in reducing the incidence of POST in paediatric patients.

**Methods:**

This randomized, double-blind, prospective study included 142 children 6–12 years of age, who were randomly assigned to receive either BH spray or control. After induction of anaesthesia, direct laryngoscope was placed and BH spray was applied to the upper trachea and vocal cord in the BH group and intubation was performed using a cuffed tube lubricated with normal saline. Intubation in the control group was performed using a cuffed tube lubricated with normal saline without any intervention. The balloon was inflated to a pressure of 20 cmH_2_O. Patients were extubated after fully awakened and transferred to the post-anaesthetic care unit (PACU), where they were examined for the presence of POST and any adverse events 30 min after arrival to the PACU. Postoperative pain was evaluated using a smartphone application.

**Results:**

Seventy-one patients were allocated to each group. The incidence of POST in the BH group did not differ from that in the control group (control: BH = 35 (49.3%): 42 (59.2%); *P* = 0.238); postoperative pain was also similar between the groups. Other complications, such as breath holding, secretions, coughing, laryngospasm and desaturation events, did not differ between the groups.

**Conclusions:**

Application of prophylactic BH spray to the vocal cords and upper trachea was not proven to reduce POST in paediatric patients.

**Trial registry:**

NCT03074968 (ClinicalTrials.gov, Feb 26, 2017).

## Background

One of the most common side effects following endotracheal intubation is postoperative sore throat (POST). The overall incidence of POST in the adult population varies from 22 to 62% [1–3], and that in paediatric population has been observed ranging from 24 to 44% [2, 4]. Some reported POST to occur at a peak incidence of 2 to 4 h after extubation in adult population whereas only limited publications regarding the incidence or peak time of POST were found among paediatric population [2, 5]. Several publications evaluated POST in children as early as 15 min since POST is worse in the early postoperative period, then decreases over time [6]. Although POST is usually alleviated over time, it lingers for 12 to 24 h, which results in significant dissatisfactions postoperatively [4].

POST is induced by direct mucosal inflammation caused by mechanical trauma with endotracheal intubation [7]. The known risk factors for POST are presence of upper respiratory tract infection, duration of anaesthesia, intubation without neuromuscular blockers, the number of intubation attempts, high cuff pressure, and the operator’s experience [2]. In particular, the use of uncuffed-endotracheal tubes and higher cuff pressure of cuffed-endotracheal tube were identified as main risk factors for POST in children [3, 4].

Several systemic reviews have suggested the use of pre-emptive local anaesthetics or anti-inflammatory drugs, such as benzydamine hydrochloride (BH), [8] lidocaine, [5, 9] ketamine, [5, 10] aspirin, [11] and dexpanthenol [12] for the prevention of POST. BH is a topical nonsteroidal anti-inflammatory drug with additional analgesic and anti-pyretic properties easily applicable to children [13]. BH is available in both topical and systemic formulations; however, due to its high volume of distribution, along with its low systemic clearance, BH is preferably used topically as an oral spray, mouthwash, or vaginal administration [13]. When topically absorbed, BH demonstrated low bioavailability with 5% or less and late peak plasma concentration occurring more than 24 h after application. This temporal residence at mucosal area benefits in treating soft tissue injury and mitigating any systemic side effects such as numbness, tingling sense of oral cavity, cough, and dry mouth [13]. Especially in alleviating POST, different topical application methods have been used, such as direct spraying at the oropharyngeal cavity, gargling, spraying the endotracheal tube cuff, lubricating at the endotracheal cuff, or in combination at both the cuff and oropharyngeal cavity [14–16]. To the best of our knowledge, there have been no clinical trials comparing the effects of BH on POST in targeting a specific population of children.

In this study, we aimed to evaluate whether spraying BH along the oropharyngeal space before intubation reduced POST in children.

## Methods

### Patient recruitment

A prospective, randomised, comparative study was conducted between March and June 2017 at Seoul National University Hospital (SNUH, Seoul, Korea). The study was approved by the SNUH Institutional Review Board (1612–061-813) and was registered at ClinicalTrials.gov (NCT03074968, Feb 26, 2017, https://register.clinicaltrials.gov/prs/app/template/EditProtocol.vm?listmode=Edit&uid=U0000Y58&ts=5&sid=S0006WDR&cx=-hdb51u). Each participant and corresponding parent were given a verbal explanation with an opportunity to ask questions about the study. Written informed consent was obtained from participants ≥7 years of age and their parents. Verbal assent was obtained from participants < 7 years of age, in addition to written informed consent from their parents. All procedures adhered to the principles of the Declaration of Helsinki.

A total of 150 children 6–12 years of age were screened, of whom 144 were ultimately enrolled. All were classified as American Society of Anaesthesiologists (ASA) physical status I-II and scheduled for elective surgery under general anaesthesia with endotracheal tube intubation. Individuals with intellectual disabilities, history of preoperative sore throat, recent upper respiratory infection, history of difficult or expected difficult airway, were excluded. In specific, difficult airway was defined as Cormack-Lehane class 3 or 4 by laryngoscopy, and ≥ 2 intubation attempts. Those who required postoperative mechanical ventilation were also excluded. Another exclusion was made depending on the type of the surgery. Ear-Nose-Throat (ENT) surgeries were limited to those not involving the airway. ENT surgeries were included only when the surgical target was limited to ear, such as myringotomy, myringoplasty, or canal wall mastoidectomy. Any surgery that invaded oropharynx, or required gastric tube insertion was not enrolled as well.

Children were prospectively screened and randomly allocated into one of the following two groups using a randomization table (online randomization software; http://www.randomisation.com): control group, and BH group. Children were enrolled by one of the investigators, while another independent investigator generated the random allocation sequence, prepared sealed opaque envelopes, opened the envelope immediately before the start of anaesthesia, and assigned participants to their respective study group.

### Anaesthetic methods

All patients arrived at the operating room without premedication and appropriately fasted according to practice guidelines from the ASA. Peripheral pulse oximetry (i.e., oxygen saturation [SpO_2_]), non-invasive blood pressure (NIBP) at 1-min intervals, and electrocardiography were monitored. N_2_O-free general anaesthesia was induced with 2–2.5 mg/kg of propofol after the 0.5 mg kg^− 1^ of 1% lidocaine administration. After loss of consciousness, the patients were manually ventilated with 8% sevoflurane and 100% oxygen at a rate of 6 L/min of fresh gas flow. For facilitation of endotracheal intubation, 0.6 mg/kg of rocuronium was administered. After confirmation of full relaxation of muscles by neuromuscular monitoring, under direct laryngoscope 4 puffs of BH spray 0.15% (Tantum, Riker Canada Inc.) 15 mg/mL was applied on the vocal cords and upper trachea in the BH group (1 puff = 175 μl) by one skilled anaesthesiologist to minimize the dose differences induced by applicator. The exact dose of BH absorbed to target was unmeasureable due to the spraying administration method. An endotracheal tube (ETT, Mallinckrodt Medcial, Athlone, Ireland) with cuff was lubricated with normal saline and inserted thereafter in both groups by paediatric anaesthesiologists with expertise and more than 2 years of experience. The use of stylet was abandoned. The size was determined using the formula: [age (in years)/4] + 3.5 and the cuff was inflated to a unifying pressure of 20 cmH_2_O using the same manometer (Cuff Pressure, Posey Co, USA) in all patients since to this date, cuff pressure of 20 cmH_2_0 is known as the standard cuff that reduce the need for tube changed without additional risk for post-extubation stridor [17]. Afterwards, auscultation was done to reassure that the cuff pressure of 20 cmH_2_O leaves air-leakage presence. During the operation additional cuff pressure measurement was not planned due to the possible mucosal irritation by air leak test measurement and manometer manipulation.

Anaesthesia was maintained using 1 minimum alveolar equivalent sevoflurane at a total flow rate of 2 L/min with 0.1–0.5 μg/mg/min of remifentanil continuous infusion. The fraction of inspired oxygen (F_I_O_2_) of inhaled gas was maintained at 40%. Minute ventilation was adjusted to maintain a partial pressure of end-tidal carbon dioxide (E_T_CO_2_) between 35 and 40 mmHg with 7 ml/kg of tidal volume. An oesophageal temperature probe (Top Probe, Meditop corporation, Republic of Korea) of 9fr was inserted immediately after intubation in a blind technique. To minimize any trauma, a smaller size of oesophageal temperature probe was used, instead of the standardized size according to children’s age. If any resistance was found, oesophageal temperature probe was not forced through the oesophagus, but the tip was placed at oral cavity, measuring oral temperature instead. In about 15 to 20 min before the completion of surgery, patient-controlled analgesia (PCA) with fentanyl (total 25 mcg/kg of fentanyl with loading dose of 1 mcg/kg, basal infusion dose of 2 mcg/kg/h, bolus dose of 0.5 mcg/kg per demand with lock out interval of 15 min) or 15 mg/kg of propacetamol was administered for postoperative pain control. The distinction between these two different postoperative analgesic practices was according to the customary dosing according to the type of the surgery.

**Fig. 1:**
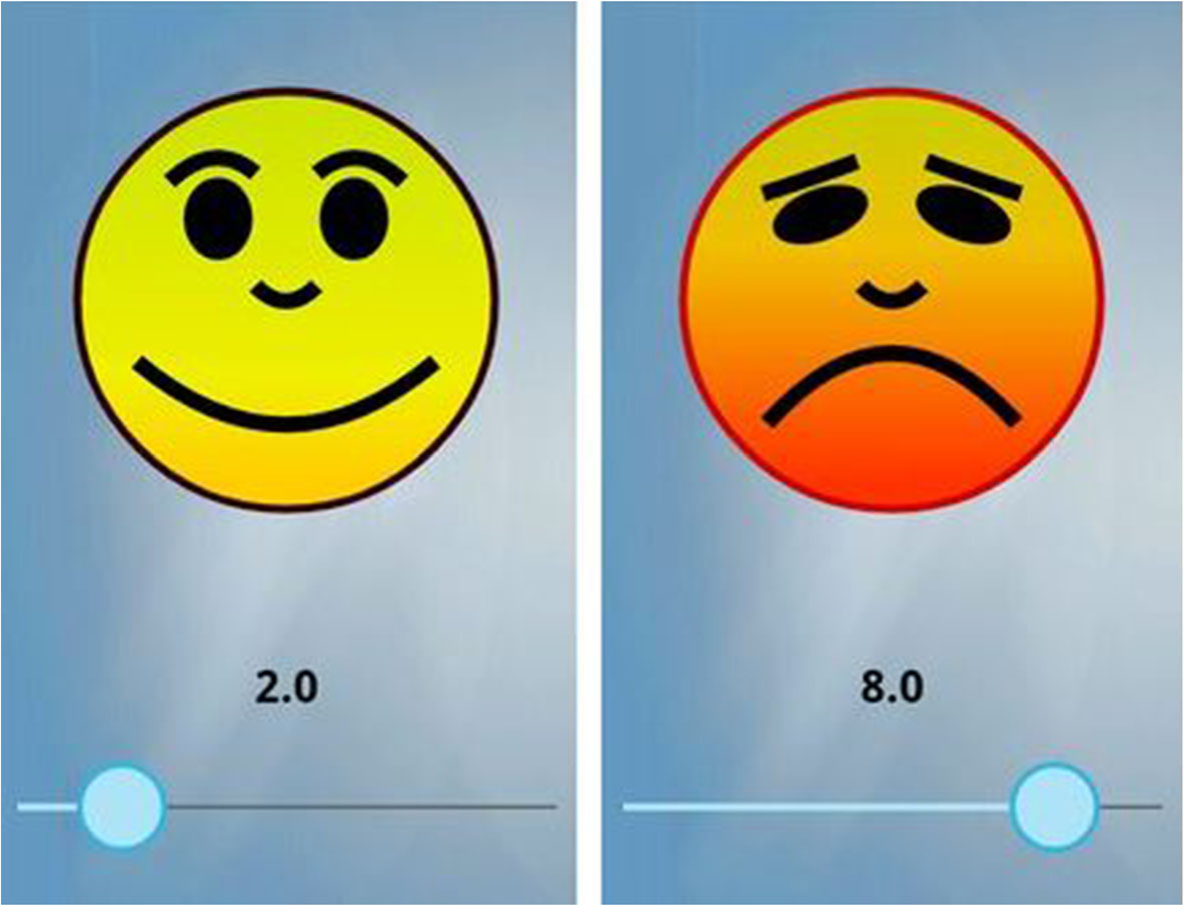
kids pain scale by *Societa di Anestesia e Rianimazione Neonatale e Pediatrica Italiana*

After the end of surgery, sevoflurane and remifentanil were discontinued and the patients were manually ventilated using 6 L/min of fresh gas flow. Antagonism of neuromuscular blockade was made with 20 mcg/kg of atropine and 40 mcg/kg of neostigmine. The patients were extubated when they maintained adequate, non-paradoxical breathing after following signs were observed; able to generate a negative inspiratory pressure > 30 cmH_2_O with spontaneous respiration; lift the head and/or limb for more than 5 s; cough forcefully after careful and gentle oral suction. During extubation, any adverse events, including breath holding for ≥20 s, coughing more than twice, excessive endotracheal secretions requiring suction, laryngospasm, or desaturation (defined as SpO_2_ < 93% [18]) were recorded. All patients were assessed at 30 min after arrival to the post-anaesthesia care unit (PACU) for severity of POST by the independent investigator who was blinded to the group allocation. To minimize any confounders of residual anaesthesia, patients were evaluated only when sufficiently awake, cooperative, and able to appropriately answer question or express their needs. The POST was evaluated using a four-point scale: (0, no sore throat; 1, mild sore throat, with complaint only on prompting; 2, moderate sore throat, with complaint without prompting; 3, severe sore throat that accompanies change in voice or hoarseness [15]. And postoperative pain was evaluated with kids pain scale (application of smartphone developed by *Societa di Anestesia e Rianimazione Neonatale e Pediatrica Italiana*, Fig. 1). Additionally, postanaesthetic emergence delirium (PAED) was verified by an independent investigator who was not formerly notified of the patient’s assigned group and the cut-off score of 12 or more was defined as PAED [19].

The primary outcome was the incidence of sore throat defined as grade > 1 on the POST four-point scale in the PACU. The secondary outcome variables were the postoperative pain, incidences of adverse events (breath holding ≥20 s, coughing ≥2 times, heavy secretion, laryngospasm, or desaturation < 93%), sore throat pain and PAED.

### Sample size estimation and statistical analysis

A previous study reported a POST incidence of 17% with BH, and 40.8% with normal saline in adults [20]. Based on this information, and the probability of a type I error (α) being 0.05 and type II error (β) being 0.05, with a statistical power at 80%, a minimum of 54 patients in each group was required according to the R program. Projecting a 20% loss in cases, 72 patients were enrolled per group.

Statistical analysis was performed using SPSS version 23.0 (IBM Corporation, Armonk, NY, USA) for Windows (Corporation, Redmond, WA, USA). The normal distribution of continuous data was evaluated using the Kolmogorov-Smirnov test, and normally distributed variables were analysed using the Student’s t test for comparison of the two groups. Categorical variables, including POST and adverse events, were analysed using Pearson’s chi-squared test (or Fisher’s exact test if expected count < 5). The results are expressed as mean ± SD with corresponding 95% confidential interval, or median (interquartile rage [25–75%]). A *P*-value < 0.05 was considered to be statistically significant. The Kruskal-Wallis test was used to compare differences in the severity of POST.

## Results

**Fig. 2:**
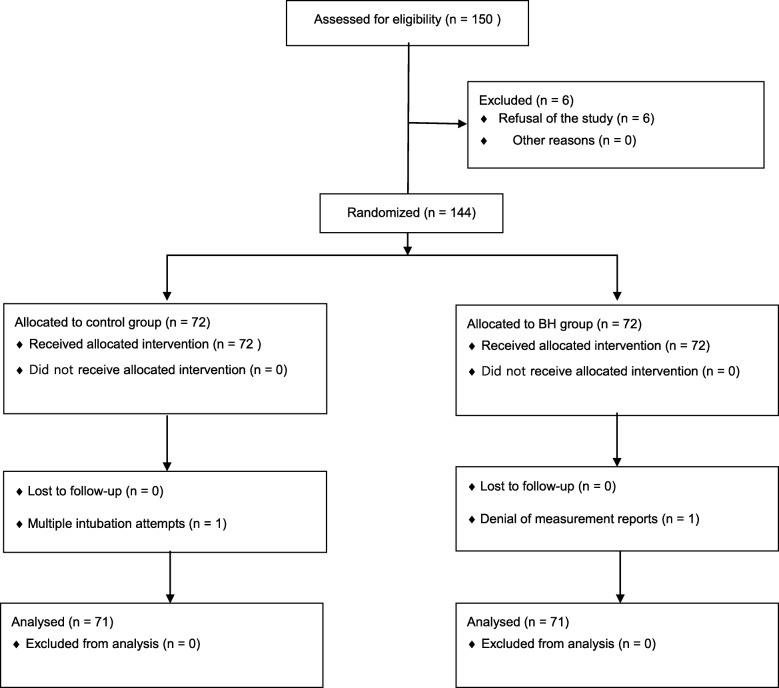
Participant flow diagram

From March to June 2017, a total of 150 children were screened, of whom 144 were recruited and considered eligible for study inclusion. As in Fig. 2, two patients, one in each group, were excluded for the following reasons. One patient from the control group dropped out due to multiple attempts at intubation (i.e., > 2 attempts). One patient from the BH group was excluded due to denial of measurement reports in the PACU. Finally, the data of the 142 patients were analysed.

Patient demographics and surgery-related parameters are shown in Table 1. There were no significant differences between the control and BH groups. In Table 2. the incidence of POST scoring > 1 demonstrated no significant difference as well. 35 (49.3%) patients in the control group experienced POST compared with 42 (59.2%) in the BH group (*P* = 0.238).

**Table 1:** Demographic variables of each group

Characteristics	Control group	BH group	*P*-value
Number (M/F)	71 (31/40)	71 (29/42)	
Age (yr)	9.3 ± 2.1	9.5 ± 1.9	0.46
Height (cm)	137.7 ± 15.7	138.0 ± 12.8	0.90
Weight (kg)	35.8 ± 13.1	37.9 ± 12.4	0.34
Anesthesia time (min)	146.3 ± 60.8	159.8 ± 72.3	0.08
Operation time (min)	106.2 ± 56.6	114.1 ± 66.3	0.16

*BH* Benzydamine hydrochloride

Results are expressed as mean ± SD with corresponding 95% confidential interval or median with interquartile rage [25–75%]

**Table 2:** Incidence of postoperative sore throat

POST		Control group (*n* = 71)	BH group (n = 71)	*P*-value
No (score = 0)		36 (50.7%)	29 (40.8%)	0.238
Yes (score **≥1**)		35 (49.3%)	42 (59.2%)	
score	1	25 (35.2%)	27 (38.0%)	
	2	7 (9.9%)	9 (12.7%)	
	3	3 (4.2%)	6 (8.5%)	

*BH* Benzydamine hydrochloride, *POST* Postoperative sore throat

All *P*-values are calculated by Chi-square test

Other adverse events related to extubation and the evaluation of postoperative pain are summarized in Table 3. All adverse events including breath holding, secretions, coughing, laryngospasm, desaturation event, and PAED did not show any significant difference between two groups. And postoperative pain evaluated with smartphone application was similar between the two groups. However, interestingly, PAED was found in more than one-half of the patients in each group. A total of 56 (78.9%) patients were evaluated with PAED in the control group, with 49 (69.0%) patients in BH group (*P* = 0.18).

**Table 3:** Incidences of adverse events

Adverse events	Control group (*n* = 71)	BH group (*n* = 71)	*P*-value
Breath holding	3 (4.2%)	2 (2.8%)	1.0
Secretion	29 (40.8%)	35 (49.3%)	0.31
Coughing	13 (18.3%)	8 (11.3%)	0.24
Laryngospasm	1 (1.4%)	0 (0.0%)	1.0
SpO_2_ < 93% within 30 min since extubation	3 (4.2%)	4 (5.6%)	1.0
PAED	56 (78.9%)	49 (69.0%)	0.18
Postoperative pain	5.3 ± 3.1	5.5 ± 3.2	0.69

All *P*-values are calculated by Chi-square or Fischer’s exact test

*SpO*_*2*_ Peripheral oxygen saturation

We reviewed the ward electric medical records for the postoperative sore throat in the recruited patients. There were no subjective significant complaints during the postoperative 24 h.

## Discussion

In our study, applying BH spray, targeting the vocal cords and upper trachea before intubation in children, did not reduce the incidence of POST compared with the control group. However, previous meta-analysis involving an adult population of randomised controlled trials (RCTs) reviewed 13 studies where reduction in the incidence of POST was reported with prophylactic BH application to the oral cavity [14].

Compared with other positive-result studies, the main difference lies in the evaluation and application time. Currently, the guideline for POST evaluation time has not been established yet. Previous RCTs evaluated POST for maximum 24 h at varying check points [15, 16]. However, we evaluated the patients at 30 min after entering the PACU, and only once. We focused on investigating POST in the immediate postoperative period, since after transferring from PACU to ward, many children were given additional analgesics by the attending physicians which is out of anaesthesiologists’ control. Moreover, unlike adult patients who are able to overtly express and verbalize their pain score after several hours, children may not be able to conceptulalize or articulate the intensity of their pain after several hours. We speculated that by focusing on the early and single time period, when the postoperative pain become the new unfamiliar concern for children, assessing score through Visual Analog Scale (VAS): kids pain scale (Fig. 1) may be effective. Yet, according to common findings of previous studies regarding BH spray (rather than gargle or gel) in adults, the highest incidence of POST occurred at 4–6 h, rather than at 0–1 h [12, 15]. The single and very early time point of our evaluation may have attributed to masking or mitigating POST by postoperative pain killers, including PCA or propacetamol, and residual anaesthetic gas. Patients might not have been sufficiently awake, cooperative, and free of delirium to answer appropriately on POST questions at this time point. In addition, the application was done at least 5 min before induction in most of the previous researches. However, we applied BH spray just before the intubation because of bitter taste of BH. This 5 min might be negligible considering that the peak effect of BH was 2–4 h.

Typically divided into the oropharyngeal space versus the ETT cuff, our study focused only on targeting the vocal cords and upper trachea. Previously, one study compared oropharyngeal space with the ETT cuff and reported that spraying BH on the ETT cuff reduces incidence and severity of POST, while spraying BH on the oropharyngeal cavity showed no additional benefit, which is consistent with our results [16]. This implies that it is difficult to reach the exact cuff inflation point only through oropharyngeal spraying. Mucosal irritation occurs at the level of ETT cuff. Applying BH directly to the cuff can definitely concentrate BH at the exact mucosal irritation, thereby significantly reducing both POST incidence and severity without further unpleasant side effects such as numbness, tingling sensation, and burning irritation, all of which children might refer to as POST [14]. While direct BH spraying easily reaches upper side of the vocal cord, it needs to overcome the vocal fold barrier to reach the cricoid level, which is the narrowest point in the paediatric airway. Thus, while BH spray may reach the trauma point in the adult airway, in paediatric patients, it may be insufficient to reach the target site.

Methodologically, differing formulations of BH, such as gel, spray or gargle, have been widely reviewed. Gel types target the tube cuff, including the endotracheal tube and supraglottic airway device tubes. Gargling covers the entire oral cavity, including the oropharynx, posterior pharyngeal wall, anterior surface of the epiglottis, and the uvula. BH gargling has demonstrated conflicting results [11, 21]. Meanwhile, spraying, as in our study, can aim both the cuff and/or oropharyngeal space, including the vocal cords and upper trachea. Spraying enables easy and fast application, with less worry about aspiration of the substances. Sprayed aerosols are smaller and widely scattered to form less tension between substances, thereby making BH widely and quickly deposited on the targeted surface area [22]. However, at the same time, this mechanism may also contribute to less convergence and lower concentration of BH to the exact airway trauma site, which is suspected to be the cricoid level in paediatric cases. Therefore, alternative aerosol delivery methods using pressurized inhalers or nebulizers may have been more effective in delivering BH to the cricoid level [22].

Additionally, the effects of normal saline as lubricant may have confounded our results, but to a low probability. In the present study, all the endotracheal tube was lubricated with normal saline immediately before intubation, and the normal saline itself may have already been sufficiently effective for the prevention of POST, thus not requiring any additional anti-inflammatory effect of BH. Normal saline can reduce the friction between ETT and airway tissues. However, the effect of such water lubrication on ETT has not been proved to significantly reduce any POST. Also, normal saline lubrication had been reported to be unrelated to diminishing POST event in diverse studies regarding adult population [2, 23, 24].

ETT cuff pressure was measured only at the time of intubation, and no measurements were taken during or at the end of surgery in our study. We found 50.7% at control group, and 40.8% at BH group reporting POST, which is higher than previously reported in the literature [1–3]. Initial ETT intubation and setting of a cuff inflation of 20 cmH_2_0 may have induced impairment of subglottic mucosal perfusion and oedema leading to higher incidence of POST in our study. We designed cuff target of 20 cmH_2_0 based on the previous report that leak at 20 to 25 cmH_2_0 ensures minimal mucosal pressure without definite air leak [25]. Although only ETT cuff pressure exceeding 30 cmH_2_0 is well-known to impair mucosal blood flow, in paediatric patients, even cuff pressure exceeding 10 cmH_2_0 has been reported to cause POST, recently [3]. Higher cuff pressures result in larger contact area and higher transmitted pressure exceeding the perfusion pressure of tracheal mucosae [3]. Since, children’s mean arterial pressure is lower than that of adult’s, cuff pressure should target inflation below 20 cmH_2_0 and continuous monitoring should be obligated to reduce unwanted cuff hyperinflation.

The present study has other several limitations, as well. First, the BH concentration at the target site was inaccurate owing to variable effect-site limiting factors, such as secretion, mucosal thickness, and spraying range, which could have influenced the results. Second, the estimation of POST severity encompasses a wide range of conditions, including pharyngitis, laryngitis, and tracheitis. Moreover, the grading even included cough and hoarseness on grading severity. It is particularly difficult to determine the cause of hoarseness because hoarseness commonly prevails in children due to children’s emergence agitation or delirium. Restless crying was often mistaken as emergence delirium, and it was difficult to define true hoarseness from heavy crying. Therefore, the severity of POST may have been overestimated in the children with occurrence of emergence delirium. At the same time, the underestimation was also easily instigated due to easy sedation and postoperative pain management using PCA or propacetamol. To compare extra mucosal irritation, checking common BH side effects, such as burning sensation, numbness and dry mouth, may have been helpful. Lastly, postoperative analgesic choice (e.g., fentanyl in PCA or bolus of propacetamol) was not randomized between the two groups because of standard protocol of administration of analgesics in our institute. Nonetheless, PCA and propacetamol incidence was similar between the two groups and different manage of postoperative pain control or use of PCA did not affect the incidence of POST in both groups.

## Conclusion

There was no benefit in using BH spray in the oropharyngeal space to reduce POST in paediatric population. However, due to the relative short period of evaluation in this study, well-designed and powered RCTs investigating the long-term effect of BH in paediatric populations must be conducted in future. Also, by comparing different application methods of BH, future studies may enable finding more accurate and practical pre-emptive local anaesthetic application within paediatric populations.

## Data Availability

The datasets used and/or analyzed during the current study are available from the corresponding author on reasonable request.

## Abbreviations

ASA: American Society of Anaesthesiologists
BH: Benzydamine hydrochloride
ENT: Ear-nose-throat
E_T_CO_2_: End-tidal carbon dioxide
ETT: Endotracheal tube
FIO_2_: Fraction of inspired oxygen
NIBP: Non-invasive blood pressure
PACU: Post-anaesthetic care unit
PAED: Postanaesthetic emergence delirium
PCA: Patient-controlled analgesia
POST: Postoperative sore throat
RCT: Rrandomised controlled trial
SpO2: Peripheral pulse oximetry
VAS: Visual analog scale
